# Specialized pro-resolving mediators in neutrophil apoptosis regulation: unlocking novel therapeutic potential in kidney diseases

**DOI:** 10.3389/fimmu.2025.1589923

**Published:** 2025-05-15

**Authors:** Yi Kang, Qian Jin, Mengqi Zhou, Huijuan Zheng, Danwen Li, Xuezhe Wang, Jingwei Zhou, Yaoxian Wang, Jie Lv

**Affiliations:** ^1^ Dongzhimen Hospital, Beijing University of Chinese Medicine, Beijing, China; ^2^ Key Laboratory of Chinese Internal Medicine of Ministry of Education, Beijing Dongzhimen Hospital, Beijing University of Chinese Medicine, Beijing, China; ^3^ Graduate School of Beijing University of Chinese Medicine, Beijing, China; ^4^ Department of Traditional Chinese Medicine, Beijing Puren Hospital, Beijing, China

**Keywords:** specialized pro-resolving mediators, neutrophils, apoptosis, efferocytosis, kidney diseases

## Abstract

Kidney diseases represent a diverse group of disorders with pathogenic mechanisms involving multiple pathological processes, including inflammation, immunity, and cell death. Neutrophils, as primary effector cells in inflammatory immune responses, participate in defending against renal infection and injury by releasing reactive oxygen species, proteases, and cytokines. However, persistent neutrophil activation is considered a crucial driver of kidney disease progression. Neutrophil apoptosis represents a critical turning point between inflammatory progression and resolution. Specialized pro-resolving mediators (SPMs) are endogenous anti-inflammatory mediators that play a critical role in resolving inflammation. They not only induce neutrophil programmed cell death and promote macrophage-mediated efferocytosis of apoptotic cells but also inhibit neutrophil infiltration and degranulation, ultimately facilitating the restoration of inflammatory microenvironment and tissue homeostasis. This review concentrates on elucidating the mechanisms by which SPMs regulate neutrophil apoptosis and systematically demonstrates their potential as novel therapeutic targets in kidney diseases.

## 1 Introduction

Kidney diseases represent a significant global public health challenge. The Global Burden of Disease study indicates that chronic kidney disease (CKD) has emerged as a leading contributor to rising global mortality rates. Once progressing to end-stage renal disease (ESRD), patients maintain poor long-term survival rates even with dialysis or transplantation (1, 2). While the precise pathogenic mechanisms of kidney diseases remain unclear, studies suggest their association with multiple factors including inflammation, autophagy, oxidative stress, and cell death. These factors may interact synergistically, ultimately leading to renal dysfunction (3, 4). Inflammation represents a crucial pathological mechanism in kidney diseases, and impaired inflammatory resolution may be a key factor driving irreversible disease progression (5). Deficiency or dysfunction of specialized pro-resolving mediators (SPMs) may result in persistent inflammation, closely associated with the chronic progression of kidney diseases (6).

Neutrophils serve as principal mediators in inflammatory responses, orchestrating defense mechanisms through the release of cytokines, chemokines, reactive oxygen species, and enzymes. While this process is essential for pathogen elimination, hyperactivation may initiate or aggravate tissue injury and chronic inflammation (7). Chronic inflammatory conditions are characterized by impaired or delayed neutrophil apoptosis (8), and the proper timing of neutrophil apoptosis has been demonstrated to be critical for inflammatory resolution across multiple inflammatory models (9). SPMs demonstrate therapeutic potential in attenuating the progression of neutrophil-mediated inflammation and chronic pathologies (including cardiovascular diseases, metabolic syndrome, and ischemia-reperfusion injury) through multiple mechanisms: reducing neutrophil recruitment, facilitating neutrophil apoptosis, and enhancing macrophage efferocytosis (10).

Based on these findings, we propose the following hypothesis: SPMs-regulated neutrophil apoptosis may influence the onset and progression of kidney diseases. This hypothesis not only explains the impact of persistent inflammation on kidney disease progression but also provides theoretical foundations for exploring novel therapeutic strategies. This review will examine in depth these mechanisms and research developments, aiming to offer new perspectives and approaches for the study and treatment of kidney diseases.

## 2 SPMs: key factors in kidney disease progression

Inflammation represents a complex defensive physiological response orchestrated by the immune system when the body encounters pathogenic infections, tissue damage, or harmful stimuli. Historically, inflammation resolution was considered a passive process, merely relying on the gradual degradation of pro-inflammatory mediators to inhibit further immune cell infiltration into damaged tissues. However, groundbreaking research by Charles Serhan’s team revealed that inflammation resolution is an actively regulated, highly programmed process involving multiple mechanisms and molecules, governed by a group of endogenous lipid mediators, which they designated as SPMs (11). Deficiency or dysfunction of SPMs may be involved in the occurrence and progression of various chronic inflammatory diseases, such as kidney disease, cardiovascular diseases, diabetes, neurodegenerative diseases, periodontitis, rheumatoid arthritis, and others (12–19). In the context of kidney disease pathogenesis and progression, SPMs demonstrate significant importance through their multifaceted actions: modulating inflammatory cell functions, maintaining homeostasis between pro- and anti-inflammatory factors, and providing protection to various renal cells, including tubular epithelial cells, glomerular mesangial cells, podocytes, endothelial cells, and fibroblasts (20).

### 2.1 Molecular identity and biological actions of SPMs

Inflammation resolution is a key phase in the regulation of the inflammatory response, with its mechanism fundamentally different from anti-inflammatory actions. From a pathophysiological standpoint, inflammation resolution begins with the peak recruitment of inflammatory cells to tissue cells, in which the body actively clears inflammatory mediators and promotes tissue homeostasis through programmed regulation (5). This process is initiated by the coordinated action of granulocytes and the monocyte/macrophage system recruited to the site of inflammation, where pro-inflammatory lipid mediators transition into pro-resolving lipid mediators, and SPMs begin to appear at the site of inflammation (21). SPMs are a class of endogenous regulatory molecules synthesized through the selective catalysis of enzymes such as lipoxygenases (LOX), cyclooxygenases (COX), and cytochrome P450 (CYP) from endogenous polyunsaturated fatty acids (PUFAs), including ω-3 fatty acids eicosapentaenoic acid (EPA), docosahexaenoic acid (DHA), docosapentaenoic acid (DPA), and ω-6 fatty acid arachidonic acid (AA) (6). Their biosynthesis depends on the cross-cellular collaboration between immune effector cells (such as neutrophils, monocytes/macrophages) and resident tissue cells (such as epithelial and endothelial cells), as these enzymes are differentially expressed in epithelial cells (15-LOX), endothelial cells (COX-2), granulocytes (5-LOX), and platelets (12-LOX) (11, 22), and are subsequently degraded by enzymes like 15-hydroxyprostaglandin dehydrogenase (15-PGDH) (23, 24). The SPMs discovered in mammals to date include four major lipids: Lipoxins (LXs), Resolvins (Rvs), Protectins (PDs), and Maresins (MaRs) (5, 23, 25), as well as non-lipid regulatory factors such as AnxA1, gaseous mediators, purines, and neuroregulatory substances (26).

**Figure 1:**
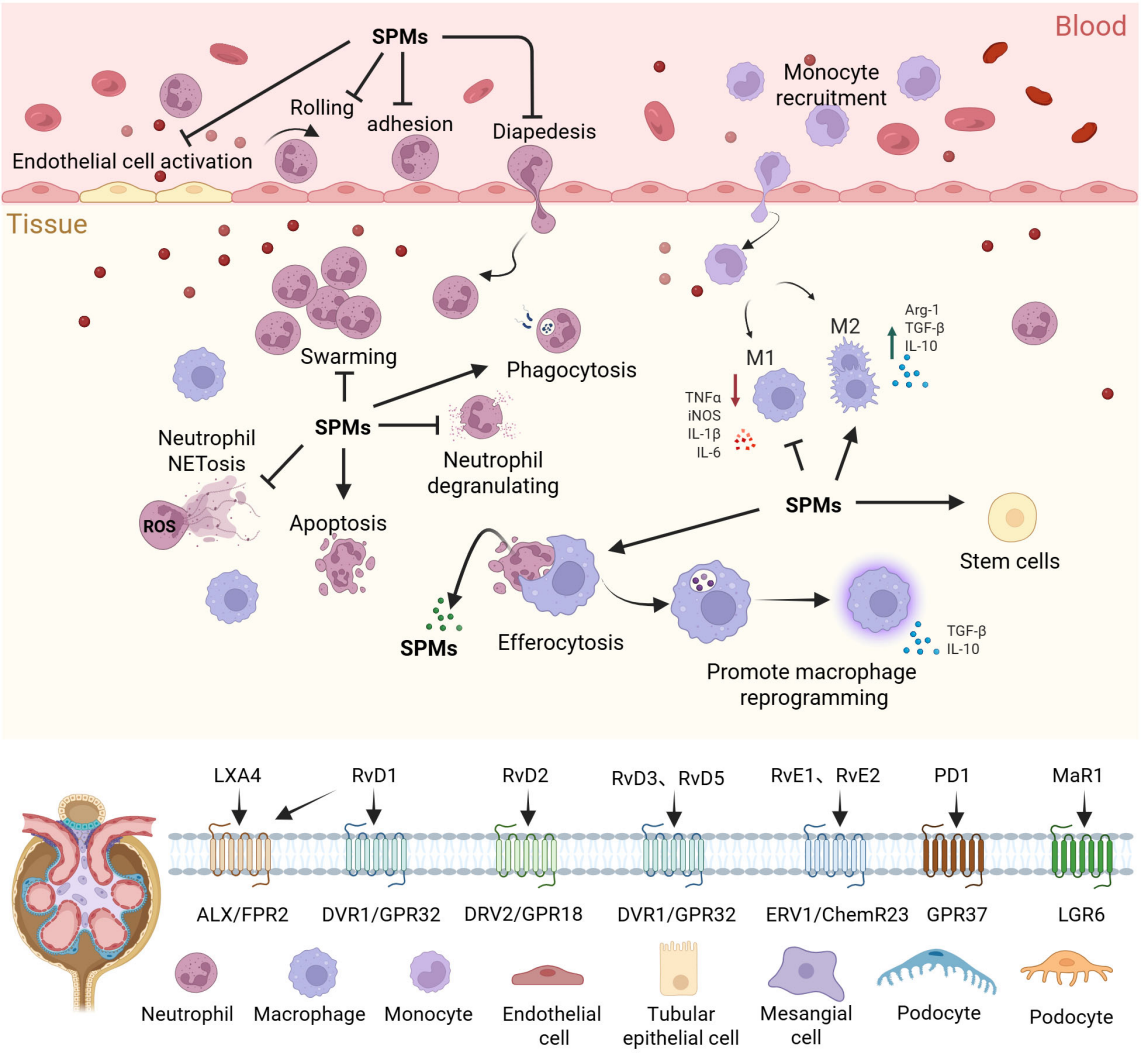
Regulation of inflammation by SPMs. SPMs regulate inflammation resolution and tissue repair through spatiotemporal dynamics. They limit the spread of inflammation by inhibiting excessive activation of endothelial cells and regulating the rolling-adhesion-transmigration cascade of neutrophils across the endothelium. Furthermore, SPMs restrict neutrophil degranulation, NETosis, and swarming, while promoting neutrophil apoptosis and enhancing phagocytosis of pathogens. SPMs also drive macrophage reprogramming, enhance efferocytosis, and facilitate the release of further SPMs. In synergy with stem cell activation, SPMs ultimately achieve the inflammation resolution and the restoration of tissue homeostasis, preventing chronic inflammatory damage. SPMs, Specialized pro-resolving mediators; TNF-α, Tumor Necrosis Factor Alpha; iNOS, Inducible Nitric Oxide Synthase; IL-1β - Interleukin 1 Beta; IL-6, Interleukin 6; Arg-1, Arginase-1; TGF-β, Transforming Growth Factor Beta; IL-10, Interleukin 10; NETosis, Neutrophil Extracellular Traps Formation; ROS, Reactive Oxygen Species.

SPMs promote inflammation resolution through G protein-coupled receptor (GPCR)-dependent signaling pathways, with different SPMs selectively activating their specific GPCRs, and their affinity for these GPCRs being in the low nanomolar range (25, 27). Although the understanding of the downstream targets following receptor activation by SPMs remains incomplete, it has been confirmed that several signaling pathways are involved, including the regulation of NF-κB, ERK, PI3K/AKT, p38 MAPK, and miRNA expression. Neutrophils, as core effector cells of the innate immune system, play a role throughout the entire process from inflammation initiation to resolution. SPMs exert pro-resolution effects by limiting neutrophil recruitment and activation, promoting neutrophil apoptosis, and facilitating macrophage-mediated efferocytosis to clear apoptotic neutrophils, among other mechanisms (12, 28, 29) (**Figure 1**). The mechanisms by which SPMs regulate inflammation resolution can be found in relevant reviews (28–31).

### 2.2 SPMs dysregulation in kidney disease pathogenesis

Inflammation, as a core pathological feature of kidney disease, when unresolved, can drive changes such as glomerulosclerosis and tubular interstitial fibrosis (32, 33), and is a key driving factor in the progression of end-stage renal disease (34, 35). Kidney regulation of the inflammatory response is disease-specific: self-limiting diseases (such as post-streptococcal glomerulonephritis) can resolve inflammation through endogenous repair mechanisms (36), while other types of kidney diseases (such as diabetic nephropathy) are often associated with chronic inflammation and sustained activation, leading to severe organ damage (37). The deficiency or dysfunction of SPMs is considered closely related to the pathogenesis of chronic inflammatory diseases (38–40), and the kidney inflammation resolution mechanism is also a process finely regulated by SPMs, the mechanisms of which are just beginning to be understood (**Table 1**).

**Table 1:** Research on SPMs in kidney diseases.

SPMs	Disease	Mechanism	Patients/Model	Key Findings	References
LXA4	lupus nephritis	Promote the generation of endogenous SPMs	SLE patients	Reduced LXA4 production is associated with acute flare-ups of lupus nephritis.	(42)
	anti-GBM nephritis	(1) Reduce neutrophil infiltration (2) Regulate IFN-γ-induced gene expression	Anti-GBM nephritis mouse model	LXA4 analogs significantly improve kidney injury	(43)
	ORG	(1) Inhibit the NF-κB and ERK/p38 MAPK pathways (2) Regulate the M1/M2 macrophage ratio in adipose tissue (3) Restore autophagy markers LC3-II/p62	ORG mouse model	Alleviate kidney morphological injury and inflammation induced by a high-fat diet	(44–47)
	DKD	(1) Inhibit glomerulosclerosis via the LXA4-ALX/FPR2 axis (2) Inhibit the release of pro-inflammatory cytokines	DKD model, mesenchymal stem cell experiment	Delay glomerulosclerosis and the progression of DKD	(48)
ANXA1	RPGN	(1) Inhibit the activation of the NF-κB p65 subunit (2) Reduce neutrophil infiltration	AnxA1 knockout mice (RPGN model)	AnxA1 deficiency exacerbates kidney injury and inflammation	(34)
	DKD	(1) Activate the AMPK/PPARα/CPT1b pathway to improve mitochondrial function (2) Regulate the MAPK pathway and activate Akt to promote survival signals	AnxA1 knockout/overexpression mice (DKD model)	Overexpression of ANXA1 alleviates proteinuria, inflammation, and fibrosis	(37, 49–51)
MaR1	DKD	(1) Activate the LGR6 receptor-cAMP-SOD2 antioxidant pathway (2) Inhibit the NLRP3 inflammasome	DKD mouse model, high glucose-induced mesangial cell experiment	Improves hyperglycemia, proteinuria, and oxidative stress	(55, 56)
	CKD	(1) Enhance macrophage phagocytic function (2) Inhibit TGF-β1-mediated mesangial cell EMT in glomeruli	Urinary testing in clinical patients (CKD staging)	Urinary MaR1 levels are significantly decreased in CKD stages 3-4	(54)

SPMs, Specialized Pro-resolving Mediators; AnxA1, Annexin A1; LXA4, Lipoxin A4; RPGN, Rapidly Progressive Glomerulonephritis; Boc-2, LXA4 Receptor Antagonist; CKD, Chronic Kidney Disease; CPT1b, Carnitine Palmitoyltransferase 1b; DKD, Diabetic Kidney Disease; EMT, Epithelial-to-Mesenchymal Transition; ESRD, End-Stage Renal Disease; FPR2, Formyl Peptide Receptor 2; GBM, Glomerular Basement Membrane; HbA1c, Hemoglobin A1c; IFN-γ, Interferon-Gamma; LC3-II, Microtubule-Associated Protein 1A/1B-Light Chain 3-II; LGR6, Leucine-Rich Repeat-Containing G-Protein Coupled Receptor 6; MaR1, Maresin 1; MAPK: Mitogen-Activated Protein Kinase; NF-κB: Nuclear Factor-Kappa B; NLRP3, NLR Family Pyrin Domain Containing 3; ORG, Obesity-Related Glomerulopathy; PMN, Polymorphonuclear Neutrophil; PPARα, Peroxisome Proliferator-Activated Receptor Alpha; SOD2: Superoxide Dismutase 2; TGF-β1, Transforming Growth Factor-Beta 1; UACR, Urine Albumin-to-Creatinine Ratio.

#### 2.2.1 Lipoxin A4

Lipoxins are the first mediators identified to specifically promote resolution (41) and have been studied in anti-glomerular basement membrane nephritis (GBM), lupus nephritis, obesity-related glomerulopathy (ORG), and DKD. In patients with Systemic Lupus Erythematosus, persistent inflammatory states may be related to a lack of endogenous SPMs, with decreased formation and release of LXA4 possibly directly associated with the development and acute flare-ups of lupus and its renal complications (42). In an anti-glomerular basement membrane nephritis (anti-GBM) mouse model, the LXA4 analog (15-epi-16-(FPhO)-LXA-Me) significantly improved renal injury in anti-GBM nephritis mice by reducing neutrophil infiltration, alleviating oxidative stress, and regulating IFN-γ-induced gene expression (43). ORG is characterized by glomerular enlargement and lipid deposition in renal cells, and is closely associated with the onset of ESRD (44). Uncontrolled inflammation is considered the pathological basis of obesity, with leukocytes from obese individuals showing impaired SPM characteristics (45). In the ORG model mouse, LXA4 injection significantly alleviated high-fat diet-induced renal morphological and functional damage, reduced the levels of pro-inflammatory cytokines and chemokines, and inhibited the activation of NF-κB and ERK/p38 MAPK pathways, with these effects significantly blocked by pretreatment with the LXA4 receptor antagonist Boc-2 (46). LXA4 and its synthetic analogs alleviate obesity-induced glomerular enlargement and mesangial matrix proliferation in mice by reducing adipose tissue inflammation, regulating the M1/M2 macrophage ratio in adipose tissue, and restoring the expression of autophagy markers LC3-II and p62 in adipose tissue (47). Notably, mesenchymal stem cells may inhibit glomerulosclerosis and pro-inflammatory cytokine release through the LXA4-ALX/FPR2 axis, thereby preventing the progression of DKD (48).

#### 2.2.2 ANXA1

ANXA1 is a 37 kDa protein, also known as lipocortin 1, initially thought to be a mediator of the anti-inflammatory effects of glucocorticoids in the host defense system. Subsequent studies have shown that it promotes nearly all mechanisms of inflammation resolution (26). Rapidly Progressive Glomerulonephritis (RPGN) and DKD are common causes of end-stage renal disease. The lack of ANXA1 may be an important factor in the progression of RPGN and DKD. Compared to wild-type mice, AnxA1 knockout mice exhibit more severe kidney injury and inflammatory cell infiltration following RPGN induction. Endogenous AnxA1 exerts renal protective effects during RPGN by inhibiting pro-inflammatory signals and neutrophil infiltration (34). Failure of inflammation resolution is a major pathological factor in the progression of DKD. In diabetic mouse models, AnxA1 deficiency exacerbates kidney damage, as evidenced by more severe proteinuria, renal inflammation, and fibrosis. Overexpression of AnxA1 significantly alleviates kidney damage, possibly through the following mechanisms: AnxA1 binds to the p65 subunit of the transcription factor NF-κB, inhibiting its activation and thereby modulating the inflammatory state (37); at the same time, AnxA1 activates the AMPK/PPARα/CPT1b pathway, improving mitochondrial function and fatty acid oxidation, which in turn reduces renal inflammation and lipotoxicity in DKD (49); it may also be involved in AnxA1’s regulation of MAPK, activating pro-survival pathways (Akt), thereby preventing cardiac and renal dysfunction (50). Clinical studies have confirmed that serum ANXA1 levels are significantly lower in DKD patients compared to diabetic patients without renal lesions (51). The expression of ANXA1 in the kidneys is increased (37), which may be a compensatory response to inflammation.

#### 2.2.3 Maresin 1

MaR1 is a potent anti-inflammatory and pro-resolving mediator synthesized by macrophages, and is the founding member of the maresins family. It effectively limits polymorphonuclear neutrophil (PMN) infiltration, enhances macrophage phagocytosis of apoptotic PMNs, promotes tissue regeneration, alleviates pain (52), regulates inflammation, and facilitates tissue repair. MaR1 has demonstrated significant protective effects in various diseases, including kidney disease, lung disease, liver disease, and vascular diseases (53). Studies have found that urinary MaR1 levels in stage 3–4 kidney disease subjects are significantly lower than in healthy individuals and stage 1–2 kidney disease patients (54). Serum MaR1 levels are significantly reduced in DKD patients and are negatively correlated with disease markers such as UACR, HbA1c, and neutrophil count. Animal experiments show that MaR1 injection improves hyperglycemia, reduces UACR, and alleviates renal pathological damage in DKD mice. It also activates the cAMP-SOD2 antioxidant pathway through the LGR6 receptor, inhibiting inflammation and oxidative stress. Cellular experiments further confirm the protective effects of MaR1. MaR1 holds promise as a predictor and therapeutic target for DKD, but its clinical application still requires further validation (55). Other studies have confirmed that MaR1 can inhibit NLRP3 inflammasome activation, reducing high glucose-induced epithelial-to-mesenchymal transition of glomerular mesangial cells mediated by TGF-β1 (56).

In conclusion, the renal protective effects of SPMs have been confirmed by multiple studies; however, there are still several scientific questions that need to be clarified. Taking DKD as an example, LXA4, ANXA1, and MaR1 are considered to play important roles in the pathogenesis of DKD. However, due to the limitations of current research, it remains unclear whether different SPMs exhibit synergistic effects. Is there an imbalance between SPMs during the disease progression, similar to what has been observed in other chronic inflammatory diseases (57)? Particularly, whether there are additive or antagonistic effects at different stages of the disease remains unsupported by systematic research evidence. Furthermore, during the resolution of inflammation, inflammatory immune cells such as neutrophils and macrophages are both the producers and effectors of SPMs. This suggests that SPMs may exert organ-protective effects indirectly through immune regulatory networks. However, current studies mostly focus on the direct protective mechanisms of SPMs on glomerular podocytes, mesangial cells, and renal tubular epithelial cells, while the analysis of the SPMs-immune cell interaction network is clearly insufficient. By drawing upon the research paradigm of ANXA1 in regulating tumor-associated macrophage efferocytosis to remodel the immune microenvironment in pancreatic cancer (58), a deeper understanding of the immune regulatory loops of SPMs and their interactions with parenchymal cells will help reveal the molecular landscape of SPMs-mediated renal protection. This will provide a theoretical foundation for developing precise therapeutic strategies based on the temporal regulation of SPMs.

### 2.3 SPMs: emerging potential and prospects for the treatment of kidney diseases

Resolution pharmacology represents an emerging therapeutic paradigm that aims to target acute and chronic inflammation through the utilization of SPMs (28). While traditional anti-inflammatory approaches are often limited by concerns about compromised host immunity and infection risks, SPMs-based therapeutic strategies not only reduce the overactivation of pro-inflammatory signals but also effectively avoid the risk of immunosuppression (26). However, endogenous SPMs are characterized by rapid degradation and high synthesis costs, which to some extent limit the potential of natural SPMs as candidate drugs for inflammatory diseases (23). In recent years, through methods such as chemical modification, several generations of SPMs mimetics (59) and their receptor agonists (60) have been successfully developed, significantly expanding the therapeutic potential of SPMs. Among them, receptor agonists such as ACT-389949 and BMS-986235 have entered Phase I clinical development (61), and several therapies based on SPMs have shown encouraging outcomes in preclinical models of both acute and CKD (**Table 2**).

**Table 2:** Mechanistic studies of SPMs in the treatment of renal diseases.

Type	Disease Model	SPMs/Mimetics	Dosing amount	Dosing time	Route of administration	Mechanistic insights	Therapeutic Outcomes	Reference
AKI	I/R	LXA4	10 μg/kg	30 mins before surgery	intravenous injection via tail vein	Inhibition of the infiltrations of neutrophils with CD45 Ly6G and macrophages with F4/80 CD11b; Inhibition of TLR4 activation; Alleviates oxidative stress and increases antioxidant enzymes;	Improve renal dysfunction and renal injury;	(62)
		MaR1	1ng/100ul	within 15 minutes after reperfusion	intravenous injection via tail vein	Inhibit the expression of TLR4 and the phosphorylation of Erk, JNK, and P38;Reduce the nuclear translocation of NF-κB; Increase the nuclear translocation of Nrf2;	Alleviate renal injury;	(63)
		protectin D1	0.4 mg/kg	given once before clamping of the renal pedicle, and a second dose after clamp release	intravenous injection via tail vein	Inhibition of neutrophil recruitment; Enhance the expression of heme-oxygenase-1;	Alleviate renal injury;	(64)
		15-epi-16-(FPhO)-LXA4-Me	15 µg	10 minutes before clamping (induction of ischemia)	Intravenous injection via the inferior vena cava	Reduction in neutrophil infiltration and decreased expression of inflammatory factors;	Alleviate renal injury;	(65)
	BSA induced	AT-RvD1	5 μg/kg	20 min prior to BSA challenge for six days	Intravenous injection	Attenuation of Iba1+ macrophage recruitment; Reducing the levels of TNF-α, IL-10, and CCL2; Reducing the levels of transforming growth factor β (TGFβ), type 3 collagen, and matrix metalloproteinase (MMP)-3 and -9;	Reduce proteinuria, urinary creatinine, and the protein-to-creatinine ratio; Alleviate renal tubulointerstitial injury;	(66)
	Paraquat induced	AT-RvD1	28 mg/kg	2 h after toxication	Intravenous injection via the inferior vena cava	Reduce the infiltration of neutrophils; Inhibit the activation of NF-κB in renal tissue and the release of inflammatory cytokines;	Improve renal dysfunction and renal injury;	(67)
	LPS induced	ATL	5 μg	2 hours before the injection of LPS	intraperitoneal injection	Reduction of Inflammatory Response; Modulation of the TLR4/MyD88/NF-κB Signaling Pathway;	Alleviate renal injury;	(68)
	CLP induced	LXA4	100 μg/kg	30 minprior to CLP	intraperitoneal injection	Inhibit NF-κB-mediated inflammatory responses; Inhibit the p53/p21 senescence pathway;	Restore renal function in rats following CLP; Enhance the survival rate of rats after CLP.	(69)
**CKD**	ADR	RvD1	4 ng/g	30 minutes or 14 days after ADR injection, continuing daily until day 28	Intraperitoneal Injection	Inhibition of 14-3-3β/synaptopodin dissociation; Reduce macrophage infiltration; Inhibit pro-inflammatory factors; Protect podocytes;	Reduce proteinuria in patients with ADR - induced nephropathy and improve renal function; Improves renal fibrosis in ADR - induced nephropathy;	(70)
	UUO	LXA4	45μg/250 g	15 minutes prior to surgery	Intravenous injection via the tail vein	Inhibit the expression of pro-inflammatory cytokines (such as TNF-α and IFN-γ) and stimulate the production of anti-inflammatory and pro-resolution cytokine IL-10; Reduce apoptosis of renal interstitial and tubular epithelial cells induced by UUO; Attenuate the activation of MAP kinases, Akt, and Smads in the damaged kidneys induced by UUO;	Alleviate renal fibrosis;	(71)
		benzo-LXA4	15 μg/250 g					
	DKD	LXA4	5 μg/kg	Twice weekly for 10 weeks (prevention study) or 16 weeks (intervention study)	intraperitoneal injections	Regulate the Egr-1 Transcriptional Network;	Mitigated diabetes-induced albuminuria, mesangial expansion, and collagen deposition.	(72)
		benzo-LXA4	1.7 μg/kg					
		Ac2-26	0.5, 1, or 2 mg/kg	Every 2 days for 10 weeks	Intraperitoneal injection	Inhibit the NF-κB signaling pathway; Reduced inflammatory cell infiltration.; Protect renal tubular epithelial cells; Protect podocytes; Promote inflammation resolution; Anti-fibrotic;	Alleviate renal injury; Reduce urinary albumin excretion;	(38, 43)
		Hr ANXA1	1 μg	Prophylactic Treatment:Week 1 to Week 13;Therapeutic Treatment: Week 8 to Week 13; During STZ Administration:Days 1–5 during STZ injection	Intraperitoneal	Inhibit pro-inflammatory and pro-fibrotic signaling pathways; Activate the Akt survival pathway;	Protects against cardiac and renal dysfunction.	(37, 49)

AKI, Acute Kidney Injury; CKD, Chronic Kidney Disease; I/R, Ischemia/Reperfusion; SPMs, Specialized Pro-resolving Mediators; MaR1, Maresin 1; PD1, Protectin D1; LXA4, Lipoxin A4; 15epi-16-(FPhO)-LXA4-Me, 15-epi-16-(Fluorophenoxy)-LXA4-Methyl ester; BSA, Bovine Serum Albumin; AT-RvD1, Aspirin-triggered Resolvin D1; RvD1, Resolvin D1; UUO, Unilateral Ureteral Obstruction; DKD, Diabetic Kidney Disease; AnxA1, Annexin A1; ATL, Aspirin-triggered lipoxin; PDX, Protectin DX; hr ANXA1, human recombinant ANXA1.

#### 2.3.1 Acute kidney injury models

In various AKI models, including ischemia-reperfusion Injury (I/R), nephrotoxic drugs, and sepsis-induced injury, SPMs treatments have demonstrated significant pro-resolving and renoprotective effects. In renal I/R models, treatments with MaR1, Protectin D1 (PD1), LXA4, and its analog 15epi-16-(FPhO)-LXA4-Me have shown to alleviate renal I/R injury through multiple mechanisms: reducing neutrophil infiltration, decreasing pro-inflammatory cytokine and chemokine expression, and inhibiting harmful lipid mediator production, thereby exerting potent anti-inflammatory and anti-oxidative stress effects (62–65). In AKI models induced by bovine serum albumin (BSA) and paraquat, RvD1 mimetics such as aspirin-triggered resolvin D1 (AT-RvD1) demonstrate therapeutic efficacy by reducing inflammatory cell infiltration in renal tissue, inhibiting NF-κB activation and inflammatory cytokine release, and activating the Nrf2 signaling pathway to upregulate antioxidant gene expression, thereby alleviating tubulointerstitial injury and fibrosis (66, 67). Furthermore, in sepsis-induced AKI models using LPS and Cecal Ligation and Puncture, pretreatment with Aspirin-triggered lipoxin (ATL) and LXA4 has shown remarkable therapeutic potential by significantly reducing serum creatinine, blood urea nitrogen, and urinary levels of kidney injury molecules and neutrophil gelatinase-associated lipocalin. These treatments also inhibited NF-κB pathway activation and decreased inflammatory cytokine levels, thereby significantly alleviating sepsis-induced AKI and improving rat survival rates (68, 69).

#### 2.3.2 CKD models

In various CKD models, including those induced by adriamycin (ADR), unilateral ureteral obstruction (UUO), and in DKD models, SPMs treatments have demonstrated significant pro-resolving and renoprotective effects. In ADR-induced nephropathy mouse models, early intervention with RvD1 has shown remarkable efficacy by reducing renal macrophage infiltration, thereby preventing ADR-induced podocyte injury, reducing proteinuria, and maintaining podocyte structural stability (70). In UUO models, LXA4 and its synthetic analog benzo-LXA4 effectively inhibit renal fibrosis progression through multiple mechanisms: suppressing pro-inflammatory cytokine expression, promoting anti-inflammatory cytokine IL-10 production, and reducing collagen deposition and cellular apoptosis in renal tissue (71). In STZ-induced Type 1 diabetes mellitus (T1DM) models, hrAnxA1 treatment administered during weeks 8–13 demonstrates therapeutic potential by attenuating the increase in proteinuria and fractional sodium excretion, while also delaying the progression of cardiac dysfunction (50). Furthermore, in STZ-induced diabetes mellitus (DM) ApoE^−/−^ mouse models, LXA4 and Benzo-LXA4 significantly ameliorate DM-induced proteinuria, glomerular expansion, and collagen deposition, demonstrating the ability to reverse established renal lesions and supporting the pro-resolving therapeutic paradigm (72). Notably, the ANAX1 mimetic Ac2–26 shows significant improvement in renal lipotoxicity in both ANAX1-/- DM mouse models (induced by STZ combined with high-fat diet) and db/db mouse models (37, 49), leading researchers to propose the ANAX1 mimetic peptide Ac2–26 as a promising therapeutic agent for DKD treatment (73).

#### 2.3.3 Positive effects of dietary SPM precursors on kidney disease

The consumption of diets rich in SPMs precursors, particularly those containing high levels of ω-3 and ω-6 PUFAs, demonstrates significant therapeutic potential in alleviating kidney disease. Fish oil, characterized by its abundance of long-chain ω-3 PUFAs, has been extensively validated through clinical trials and experimental studies to enhance glucose and lipid metabolism while reducing oxidative stress and inflammatory responses (74–77). Notably, in CKD patients, ω-3 PUFA supplementation exhibits a strong correlation with elevated circulating SPMs levels (78). In an ESRD mouse model utilizing LDLr/ApoB^-/-100/100^ mice fed with a high-fat high-sucrose (HFHS) diet, dietary supplementation with long-chain ω-3 PUFAs resulted in increased Protectin DX (PDX) levels, effectively preventing ESRD and cardiac dysfunction. PDX-treated mice demonstrated significant improvements in renal fibrosis and glomerular expansion, alongside preserved cardiac function, manifested through enhanced ejection fraction, maintained heart rate, and increased cardiac output—effects potentially independent of glycemic regulation (79). Furthermore, in the 5/6 nephrectomy CKD model, ω-3 supplementation effectively attenuated oxidative stress, inflammation, and fibrotic responses in the remnant kidney (80). Extensive clinical and experimental evidence has established a robust connection between AKI and CKD (81, 82). Considering the pivotal role of SPMs in both acute and chronic kidney diseases, we posit that the progression of these conditions is intrinsically linked to unresolved primary renal injury and persistent inflammation. Neutrophils, serving as central mediators of inflammatory responses, can initiate or amplify tissue damage and chronic inflammation when aberrantly activated. The mechanistic underpinnings of how delayed or impaired neutrophil apoptosis contributes to kidney disease pathogenesis warrant further investigation.

## 3 Neutrophil apoptosis: a critical determinant in renal homeostasis and disease progression

Neutrophils are a crucial part of the innate immune system, constituting 50%-70% of human blood leukocytes. As primary responders during the initial phase of inflammation, these cells rapidly infiltrate inflamed tissues, serving as critical mediators in host defense against infection. Neutrophils exhibit remarkable plasticity in adapting to their microenvironment. Mature neutrophils are characterized by their relatively short half-lives in blood circulation and various tissues, though their longevity varies significantly across different tissue types (83) and can be modulated by local inflammatory signals (84, 85). Following the completion of their immune functions, neutrophils undergo programmed cell death (apoptosis) and are subsequently recognized, phagocytosed, and eliminated by macrophages. When neutrophil apoptosis is delayed or apoptotic neutrophils are not efficiently cleared, these cells continue to release substantial quantities of lysosomal enzymes, Reactive Oxygen Species (ROS), and inflammatory mediators, resulting in tissue damage and inflammatory amplification. In the context of kidney diseases, delayed neutrophil apoptosis can lead to persistent or chronic inflammation, subsequently exacerbating renal tissue injury (86–88).

### 3.1 Molecular orchestration of neutrophil apoptosis

The human bone marrow generates approximately 10^11^ neutrophils daily, with neutrophil apoptotic duration demonstrating remarkable plasticity. This temporal regulation can be extended to enhance microbial pathogen elimination or abbreviated to facilitate inflammation resolution, representing a critical mechanism in maintaining circulating neutrophil homeostasis and effectively resolving inflammatory responses (89). The process of neutrophil apoptosis is orchestrated through an intricate network of pro-survival and pro-apoptotic signaling pathways.

#### 3.1.1 Core molecular mechanisms and regulatory pathways of neutrophil apoptosis

Neutrophil apoptosis takes place through two distinct mechanisms: the extrinsic death receptor pathway and the intrinsic mitochondrial pathway. Cysteinyl aspartate specific protease (Caspase), expressed as multiple cysteine family proteases in neutrophils, functions as the central executor of both apoptotic pathways (90). The extrinsic pathway begins with the activation of death receptors on the neutrophil surface. When ligands bind to these receptors, they initiate the recruitment of adaptor molecules and death domains, which ultimately result in the formation of death-inducing signaling complexes. These complexes interact with pro-caspase-8, facilitating its conversion to caspase-8 and subsequent activation of caspase-3, ultimately executing apoptosis through the activation of Caspase-Activated DNase (CAD) (91–94). In the intrinsic pathway, the dynamic interplay between Bcl-2 family members, including pro-survival proteins (Mcl-1, A1, Bcl-x) and pro-apoptotic proteins (Bad, Bax, Bak), is mediated through crucial dimerization processes. During apoptosis, pro-apoptotic proteins Bid and Bax translocate to mitochondria, enhancing outer membrane permeability and facilitating cytochrome c release. The released cytochrome C then binds to apoptosis protease-activating factor-1 (Apaf-1), triggering its oligomerization and subsequent activation of caspase-9 (95). This activation cascade proceeds through caspase-9-mediated activation of caspase-3, ultimately culminating in cellular apoptosis (96). This sophisticated process is intricately regulated by multiple signaling pathways and genes, including MAPK/ERK, PI3K, NF-κB, and JAK/STAT (97).

#### 3.1.2 Cellular interactions and inflammatory mediator regulatory networks in delayed neutrophil apoptosis

**Figure 2:**
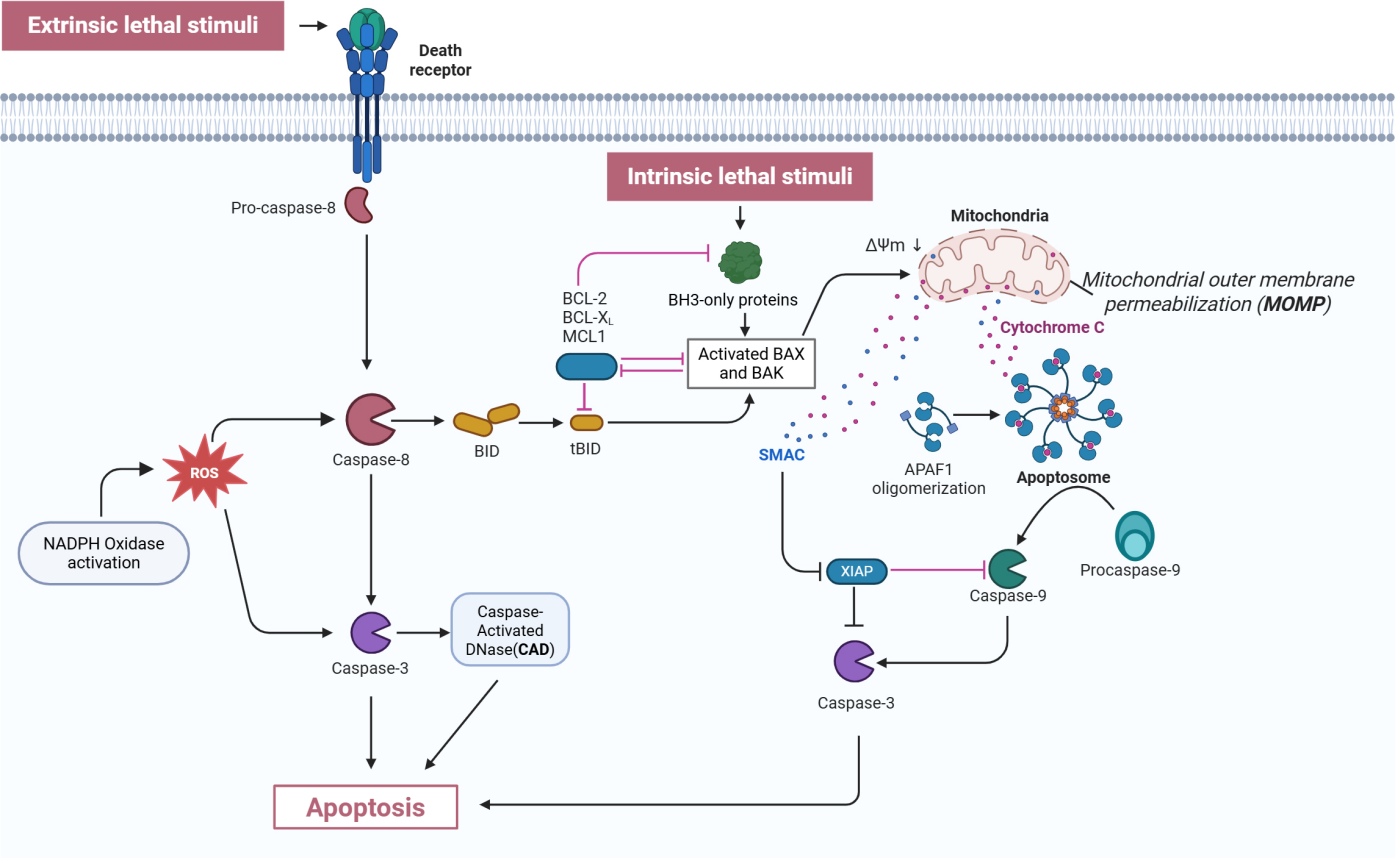
Mechanism of neutrophil apoptosis. This figure depicts both the extrinsic and intrinsic apoptotic pathways. In the extrinsic pathway, death receptors are triggered by external lethal signals, resulting in the activation of pro-caspase-8, which is then converted into active caspase-8. Activated caspase-8, on one hand, directly activates caspase-3, and on the other hand, cleaves BID protein into tBID. Simultaneously, NADPH oxidase is activated to generate ROS, which further activate caspase-3 and CAD. In the intrinsic pathway, intrinsic lethal stimuli activate BH3-only proteins, a process which is inhibited by BCL-2, BCL-XL, and MCL1. BH3-only proteins activate BAX and BAK, resulting in MOMP and the release of cytochrome C. The released cytochrome C binds to APAF1, forming the apoptosome, which then activates procaspase-9 into caspase-9. This process is inhibited by XIAP, while SMAC counteracts XIAP. Ultimately, caspase-9 activates caspase-3, triggering apoptosis. Both pathways ultimately execute the apoptosis program through the activation of effector caspase-3, illustrating the complex regulatory network of the apoptosis signaling pathway. CAD: Caspase-activated DNase; ROS, Reactive oxygen species; XIAP, X-linked Inhibitor of Apoptosis Protein; tBID, truncated BID; ΔΨm, mitochondrial membrane potential; APAF1, Apoptotic protease activating factor 1; MOMP, mitochondrial outer membrane permeabilization; SMAC, Second mitochondria-derived activator of caspases. This Figure adapted from Ona (2020); created with BioRender (biorender.com).

Delayed neutrophil apoptosis is governed by both direct cellular interactions and indirect effects of soluble inflammatory mediators. The direct cellular interactions involve macrophages, NK cells, and endothelial cells, while the indirect effects are mediated through pro-inflammatory factors and SPMs. Macrophages orchestrate neutrophil recruitment to inflammatory sites through chemokine release and produce granulocyte-macrophage colony-stimulating factor (GM-CSF), which extends neutrophil survival (98). Recent investigations have revealed that during the non-phagocytic resolution phase, macrophage-derived IFN-β activates the STAT3 signaling pathway to promote neutrophil apoptosis, enhance macrophage phagocytosis of apoptotic neutrophils, and facilitate macrophage reprogramming toward anti-inflammatory and pro-resolving phenotypes. This creates a positive feedback loop promoting inflammation resolution, suggesting that delayed neutrophil apoptosis may result from insufficient macrophage IFN-β production (99). NK cells contribute to neutrophil survival through GM-CSF and IFN-γ secretion (100), while neutrophils reciprocally stimulate NK cell IFN-γ production, establishing a positive feedback loop under inflammatory conditions (101). Endothelial cells extend neutrophil lifespan through paracrine GM-CSF secretion (102). In addition to GM-CSF, low concentrations of TNF-α prolong the survival of cultured neutrophils (103). The deficiency or dysfunction of SPMs represents another significant factor in delayed neutrophil apoptosis (104) (**Figure 2**). Moreover, recent studies have demonstrated that some neutrophils can undergo reverse migration from inflammatory tissues back into circulation, a process that enhances their anti-apoptotic properties and consequently exacerbates tissue damage (105).

### 3.2 Aberrant neutrophil apoptosis in kidney diseases

#### 3.2.1 Close association between kidney disease risk factors and neutrophil apoptosis

Hyperlipidemia, hyperglycemia, aging, and cardiovascular diseases (CVD) are common risk factors for kidney disease, and they are intricately linked to chronic inflammation and consequent tissue damage. These metabolic disturbances induce the production of pro-inflammatory neutrophils, perpetuating chronic inflammation (106). In the context of CKD and hemodialysis patients, high-density lipoprotein (HDL) demonstrates significant inhibitory effects on neutrophil apoptosis, potentially mediated through the activation of PI3K and Extracellular signal-Regulated Kinase (ERK) signaling pathways in Polymorphonuclear Neutrophils (PMN) (107). In type 2 diabetes mellitus (T2DM) patients, neutrophil apoptosis rates exhibit a significant correlation with Hemoglobin A1c (HbA1C) levels. The chronic hyperglycemic state suppresses natural neutrophil apoptotic processes, potentially exacerbating tissue damage and elevating the risk of microvascular complications (108). A distinct neutrophil subpopulation characterized by anti-apoptotic and pro-inflammatory features has been identified in T2DM patients, suggesting that reduced neutrophil apoptosis may contribute to the risk of CKD development in these patients (109). Notably, neutrophil count demonstrates a strong correlation with the occurrence of autoimmune DKD (110), and elevated neutrophil numbers may serve as an independent predictor of CKD progression in diabetic patients (111). Furthermore, under renal inflammatory conditions, pro-inflammatory factors, including GM-CSF and IL-8, can delay neutrophil apoptosis through multiple pathways (112). Aging is also accompanied by a decrease in the levels of SPMs (113, 114). With advancing age, neutrophil phagocytic activity, cytokine production, and reactive oxygen species (ROS) generation all decline, while the production of pro-inflammatory cytokines increases, leading to excessive formation of neutrophil extracellular traps (NETs) and exacerbating chronic inflammation (115). Inflammation is considered a key driver of many age-related kidney diseases (114). In CVD, impaired clearance of apoptotic neutrophils (116) and the release of activated NETs are important contributors to the inflammation in both CVD (117) and kidney disease (118). These findings highlight that neutrophil apoptosis and dysfunction serve as common hubs for kidney damage induced by metabolic disturbances, aging, and cardiovascular diseases.

#### 3.2.2 Pathological mechanisms of neutrophil apoptosis in various kidney diseases

Neutrophil apoptosis serves as a critical determinant in kidney recovery, with its dysregulation potentially accelerating renal injury and disease progression (**Table 3**). Studies utilizing Bax gene knockout bone marrow chimeric mice during IRI recovery have demonstrated that impaired neutrophil clearance results in delayed functional and tissue recovery, elevated cytokine levels, and accelerated renal fibrosis (119). In antineutrophil cytoplasmic antibody-associated vasculitis (AAV), delayed neutrophil apoptosis emerges as a crucial factor in secondary renal injury (120). Renal biopsies from ANCA-positive RPGN patients reveal an imbalance between neutrophil apoptosis and proliferation, favoring cellular proliferation over apoptosis-mediated inflammation resolution (121). In anti-glomerular basement membrane glomerulonephritis, neutrophil-dependent kidney injury represents a fundamental pathogenic mechanism. Notably, CD44, a molecule involved in human monocyte recognition of apoptotic neutrophils, demonstrates the capacity to rapidly induce neutrophil apoptosis *in vitro* while inhibiting neutrophil-dependent kidney injury *in vivo* (122). In Poststreptococcal Glomerulonephritis (PSGN), neutrophil and macrophage infiltration and activation constitute primary drivers of renal injury (123). While PSGN typically follows a self-limiting course, some patients progress to chronic renal failure, potentially due to inadequate induction of cellular apoptosis, particularly in neutrophils (124). In lupus nephritis, insufficient clearance of apoptotic neutrophils leading to secondary necrosis and autoimmune responses may significantly contribute to disease activity (125). Research in immune complex glomerulonephritis (ICGN) models has revealed that less than 20% of neutrophils undergo apoptosis within glomeruli, with the majority exhibiting reverse migration into circulation (105). This reverse migration process enhances neutrophils’ anti-apoptotic characteristics, consequently exacerbating tissue damage (126). Clinical investigations have further demonstrated that neutrophil dysfunction and delayed apoptosis, induced by elevated serum immunoglobulin light chain (IgLC) levels in renal failure patients, represent significant factors in uremic patients’ susceptibility to infections. These infections often constitute critical determinants of disease progression and mortality in ESRD patients (127).

**Table 3:** Pathological roles and intervention strategies of aberrant neutrophil apoptosis in kidney diseases.

Disease Context	Key Findings	Mechanistic Insights	Clinical Relevance	Therapeutic Potential	References
Ischemia-Reperfusion Injury	Delayed neutrophil clearance in Bax knockout chimeric mice	Apoptotic pathway defect (Bax deficiency) → persistent inflammatory microenvironment	Accelerated renal fibrosis and delayed functional recovery	Targeting Bcl-2 family pro-apoptotic proteins	(119)
ANCA-associated vasculitis	Imbalance between neutrophil apoptosis and proliferation in kidney	Anti-neutrophil antibodies interfere with apoptotic signaling → enhanced NETosis	Increased risk of crescentic glomerulonephritis progression	Induction of specific apoptotic pathways (e.g., CD44 activation)	(120, 121)
Anti-GBM GN	CD44-mediated neutrophil apoptosis inhibits renal injury	CD44-mediated phagocytic recognition → promotion of apoptotic signal transduction	Modulation of acute kidney injury severity	Development of CD44 agonists	(122)
Post-Streptococcal GN	Delayed neutrophil apoptosis associated with chronicity	Pathogen-associated molecular patterns (PAMPs) → formation of apoptosis-resistant phenotype	Risk of chronic renal failure transformation	Risk of chronic renal failure transformation	(123, 124)
Lupus Nephritis	Defective clearance of apoptotic neutrophils leading to secondary necrosis	Impaired MerTK-mediated efferocytosis → autoantigen release	Disease activity biomarker	Therapeutic strategies to enhance efferocytosis	(125)
Immune-Complex GN	Reverse migration of neutrophils (80%) enhancing anti-apoptotic properties	Aberrant CXCR2/CXCL1 chemokine axis → activation of bone marrow homing signals	Mechanism of recurrent kidney injury	Blocking reverse migration signaling pathways	(105, 126)
End-Stage Renal Disease	Uremic serum-induced delay in neutrophil apoptosis	IgLC accumulation → mitochondrial pathway inhibition → immunosenescence phenotype	Increased infection-related mortality	Adsorptive removal of pathogenic IgLC	(127)

ANCA, Anti-Neutrophil Cytoplasmic Antibodies; GBM, Glomerular Basement Membrane; NETosis, Neutrophil Extracellular Traps formation; PAMPs, Pathogen-Associated Molecular Patterns; CXCR2, C-X-C Chemokine Receptor Type 2; CXCL1, C-X-C Motif Chemokine Ligand 1; MerTK, Tyroprotein Kinase Mer; IgLC, Immunoglobulin Light Chains.

## 4 Synergistic therapeutic: inflammatory resolution mediators modulating neutrophil apoptosis in kidney disease

Neutrophil apoptosis serves as the initiating signal for inflammation resolution and marks a critical turning point between persistent inflammation and its resolution (106, 128). SPMs exhibit multiple regulatory functions, including the inhibition of neutrophil recruitment and activation while promoting apoptosis and efferocytosis. The therapeutic induction of cellular apoptosis has emerged as a central focus in inflammation resolution research.

### 4.1 SPMs and neutrophil apoptosis

**Figure 3:**
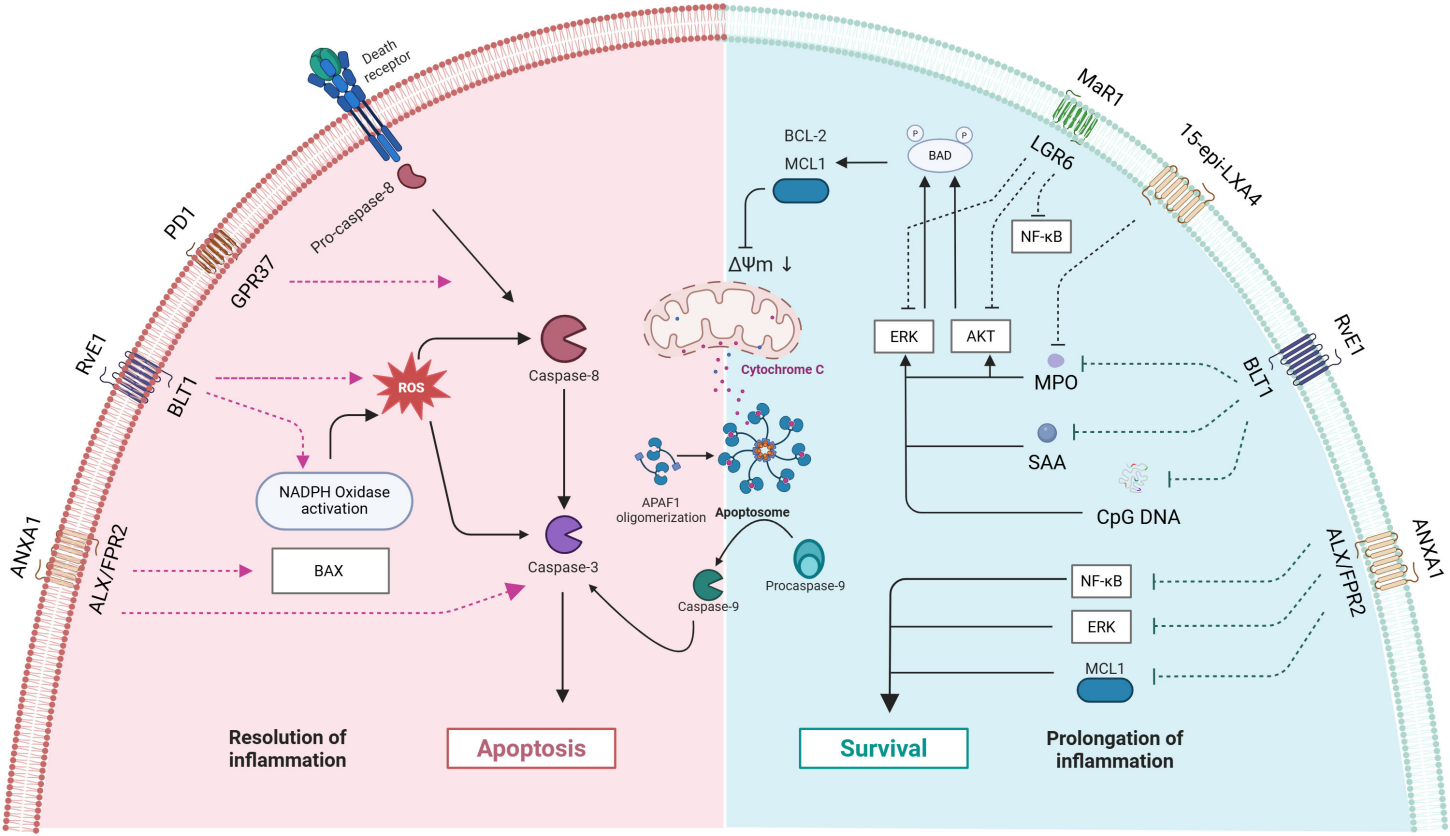
Mechanisms by which mediators of inflammation resolution regulate neutrophil apoptosis and survival. The image is divided into two regions: the left side (pink area) represents the pathway of inflammation resolution, while the right side (blue area) represents the pathways of cell survival and inflammation prolongation. The blue module depicts pro-survival mechanisms, including BCL-2 family anti-apoptotic proteins, survival signaling pathways (AKT, ERK, NF-κB), and the inflammation-prolonging states they mediate. The red module illustrates pro-apoptotic mechanisms, encompassing caspase cascade activation and mitochondrial apoptosis pathways. The interactions between various receptors and signaling molecules collectively determine the ultimate cell fate and the progression of inflammation. PD1, Protectin D1; GPR37, G Protein-Coupled Receptor 37; ANXA1 - Annexin A1; ROS, Reactive Oxygen Species; ΔΨm, mitochondrial membrane potential: APAF1, Apoptotic Protease Activating Factor 1; MPO, Myeloperoxidase; SAA, Serum Amyloid A; CpG DNA, Cyclodextrin-Glycosylated DNA.

Growing evidence underscores neutrophil apoptosis as a crucial determinant of inflammatory response outcomes and a promising therapeutic target (128–131). Notably, endogenous lipid mediators, such as LXA4 and Cyclin-Dependent Kinase (CDK) inhibitors (132), demonstrate the ability to promote neutrophil apoptosis in animal models while enhancing acute inflammation resolution. SPMs’ regulation of neutrophil apoptosis operates through multiple beneficial mechanisms: preventing excessive neutrophil activation and subsequent inflammatory exacerbation, avoiding secondary necrosis of apoptotic neutrophils and the associated release of tissue-damaging toxic contents, and promoting macrophage metabolic reprogramming (**Figure 3**).

#### 4.1.1 Promote the release of SPMs

Neutrophils play a pivotal role in inflammation resolution as key immune cells involved in SPMs transcellular synthesis and express various SPMs receptors. The process of neutrophil apoptosis is characterized by the production of multiple SPMs (20, 23, 128, 129, 133, 134), including crucial intracellular proteins such as AnxA1 and calgranulins. AnxA1, a well-established pro-resolving factor, exhibits distinct distribution patterns in neutrophils: primarily cytoplasmic in resting cells, with significant presence in cytoplasmic granules and other organelles (135). Upon cellular activation and endothelial adhesion, AnxA1 undergoes rapid translocation to the cell surface (136), where it forms complexes with proteinase 3 and PLSCR1, facilitating phosphatidylserine externalization during apoptosis (137). Through interaction with formyl peptide receptor 2 (FPR2/ALX) on the membrane, it modulates leukocyte adhesion and migration (138, 139). The soluble AnxA1 released during apoptosis demonstrates multiple pro-resolving functions: inducing neutrophil apoptosis, regulating monocyte recruitment, and enhancing macrophage-mediated clearance of apoptotic cells. In the resolution phase of inflammation, neutrophils undergo a remarkable metabolic shift in their arachidonic acid pathway, transitioning from the production of pro-inflammatory leukotriene B4 to anti-inflammatory lipoxin A4 (140).

#### 4.1.2 Inhibit inflammatory damage caused by cell necrosis

Apoptosis is considered an ideal form of cell death as it effectively prevents inflammation spread by containing cellular contents within membranes. However, during inflammatory responses, other cell death mechanisms (autophagy, necrosis, and NETs deposition) may also be activated (141). Non-apoptotic cell death can promote inflammatory responses through delayed clearance processes, as described in NETosis (142). Studies have shown that SPMs (including AnxA1, Resolvin E1, Protectin D1, Maresin 1, NO, 15-epi-LXA4, 17-epi-RvD1, etc.) participate in dual regulation of pro-apoptotic and anti-apoptotic pathways through binding to receptors such as ALX/FPR2, ChemR23, and BLT1. AnxA1 regulates both natural and glucocorticoid-induced inflammation resolution by inducing neutrophil apoptosis, activating pro-apoptotic pathways (including Bax and caspase-3 activation), and inhibiting survival pathways (including suppression of Mcl-1, ERK1/2, and NF-κB) (143). RvE1 induces apoptosis by promoting neutrophil phagocytosis, enhancing NADPH oxidase-generated ROS and activation of caspase-8 and caspase-3. Through binding to BLT1 receptor, it inhibits anti-apoptotic signals produced by inflammatory mediators such as myeloperoxidase (MPO), serum amyloid A (SAA), and bacterial DNA (CpG DNA) (which inhibit neutrophil apoptosis by activating ERK and Akt signaling pathways and increasing Mcl-1 expression), disrupts mitochondrial transmembrane potential (ΔΨm), thereby promoting neutrophil apoptosis (144). Protectin D1 promotes neutrophil apoptosis by enhancing caspase-dependent pathways, thereby facilitating inflammation resolution in LPS-induced acute lung injury mouse models (145). Maresin 1 promotes resolution of LPS-induced acute lung injury inflammation by inhibiting AKT, ERK, and p38 phosphorylation, downregulating Mcl-1 and Bcl-2 expression, and ultimately mediating neutrophil apoptosis through caspase-3 activation (146). In cerebral ischemia-reperfusion models, macrophage-derived maresin 1 inhibits NF-κB pathway activation, and suppressing production of pro-inflammatory mediators such as IL-1β, and TNF-α (147). Through the ALX/FPR2 receptor, 15-epi-LXA4 and 17-epi-RvD1 can antagonize CpG DNA and mitochondrial DNA (mtDNA) signals, maintain C5aR expression, restore impaired phagocytosis, and improve bacterial clearance while promoting phagocytosis-induced neutrophil apoptosis, thereby alleviating E. coli-induced lung injury (148). 15-epi-LXA4 facilitates human neutrophil apoptosis by inhibiting MPO-induced extracellular signal-regulated kinase and Akt-mediated Bad phosphorylation, decreasing the expression of the anti-apoptotic protein Mcl-1, and downregulating β2 integrin Mac-1 signaling. Furthermore, treatment with 15-epi-LXA4 effectively promotes the resolution of inflammation by accelerating neutrophil apoptosis and decreasing the release of inflammatory mediators (149).

#### 4.1.3 Promoting clearance of apoptotic neutrophils

The efficient phagocytosis of apoptotic neutrophils, a process termed efferocytosis, represents a crucial endpoint in cellular apoptosis that effectively minimizes the release of cytotoxic contents and subsequent tissue injury. Macrophage recognition of apoptotic cells initiates feedback mechanisms that program these cells toward an enhanced efferocytosis phenotype (106). Significantly, efferocytosis promotes macrophage polarization toward anti-inflammatory and pro-homeostatic phenotypes (150). Macrophage lysosomes process phagocytosed apoptotic cells, liberating metabolic substrates, including lipid components from apoptotic cell membranes and SPMs. These substrates directly influence macrophage polarization potential and modulate their functional properties (151–153). During efferocytosis, neutrophils orchestrate their own clearance through the presentation of “find-me” and “eat-me” signals. The “find-me” signals serve as chemoattractants for scavenger cells, while the “eat-me” signals, expressed on the apoptotic cell surface, initiate the phagocytic process. AnxA1, one of several molecular mediators involved in macrophage recognition of apoptotic cells (106), undergoes redistribution to the cell membrane during neutrophil activation or apoptosis, facilitating macrophage recognition and subsequent clearance (154–156). 17R-RvD1 enhances macrophage-mediated phagocytosis of damaged or apoptotic erythrocytes and polymorphonuclear leukocytes while simultaneously suppressing NF-κB activation and reducing the expression of inflammatory cytokines and vascular activation markers, thereby attenuating inflammation and renal vascular dysfunction in patients with sickle cell disease (157). Resolvin E1 and PD1 enhance macrophage efferocytosis through activation of cellular ChemR23 and GPR37 receptors (158–161). ChemR23 receptor activation promotes both macrophage efferocytosis and neutrophil apoptosis, accelerating the resolution of acute inflammation and triggering inflammatory resolution in chronic colitis models (162). The anti-inflammatory mediators released during macrophage efferocytosis, particularly TGFβ and IL-10, likely serve as principal drivers of M2 polarization (163).

### 4.2 Therapeutic approaches targeting SPMs and neutrophil apoptosis

While research examining the role of SPMs in regulating neutrophil apoptosis in kidney disease remains limited, existing studies have identified pathological conditions characterized by impaired SPMs-mediated regulation of neutrophil apoptosis. Recent investigations have revealed that CKD patients exhibit increased intracellular AnxA1 expression in neutrophils, coupled with decreased membrane-bound and secreted AnxA1, suggesting compromised utilization of AnxA1’s anti-inflammatory and pro-resolving functions (164). AnxA1 deficiency, resulting in delayed neutrophil apoptosis and impaired macrophage efferocytosis, may contribute to persistent and exacerbated inflammatory responses in glomerulonephritis (165). In AAV-induced renal injury, delayed neutrophil apoptosis represents a critical pathogenic factor, where increased AnxA1 localization on apoptotic cell membranes, forming membrane protein platforms with proteinase 3 and phospholipid scramblase 1, may interfere with the normal clearance of apoptotic neutrophils (120). Studies utilizing RPGN models in AnxA1-deficient and wild-type mice have demonstrated AnxA1’s renoprotective effects through reduced neutrophil infiltration and enhanced neutrophil apoptosis (34). In investigations of cardiorenal syndrome following myocardial infarction, RvD1 administration effectively modulated inflammatory responses by promoting neutrophil clearance and increasing reparative macrophages, thereby limiting both cardiac and renal inflammatory injury (166).

Regulating neutrophil apoptosis has been shown in various diseases (e.g., cystic fibrosis (CF), acute lung injury, acute respiratory distress syndrome, myocardial infarction) to protect the body from damage by reducing NET levels and decreasing the release of pro-inflammatory mediators (167–171). Notably, similar to kidney diseases, the biosynthesis of SPMs, such as ANXA1, NO, and LXA4, is reduced in CF patients, and the levels of SPMs in the respiratory tract are correlated with lung function in CF patients (172). Experimental studies have confirmed that RvE1 can induce neutrophil apoptosis through phagocytosis and alleviate the exacerbation of acute lung inflammation caused by enhanced anti-apoptotic signals (144). In the context of myocardial infarction, defects in the synthesis of SPMs (LXA4, RvD1) are clearly associated with poor prognosis (173, 174). SPMs are potential targets for regulating neutrophil apoptosis, and the modulation of SPMs to control neutrophil apoptosis could facilitate the resolution of inflammation, offering promising therapeutic avenues for the aforementioned diseases.

Although “Resolution pharmacology” research primarily relies on *in vitro* cellular and *in vivo* disease models, stable SPMs analogs have demonstrated therapeutic efficacy in clinical trials for asthma and eczema (175, 176). Drug-loaded nanoparticles and formulations demonstrate the ability to selectively target inflammatory neutrophils *in situ* and induce neutrophil apoptosis through intracellular delivery, thereby mitigating inflammatory injury (177–179). Nano-pro-resolving medicines enhance human macrophage phagocytosis and bacterial/debris clearance, significantly abbreviating inflammation resolution time and reducing PMN infiltration, thus suppressing inflammation and promoting tissue repair (113, 180, 181). This suggests that nanotechnology may have significant translational medical value for targeting SPMs regulatory systems in inducing neutrophil apoptosis, offering new hope for developing precise strategies to modulate the inflammatory microenvironment in kidney diseases. However, the feasibility of this therapy needs to be carefully evaluated. Nanomedicines still face technical challenges such as premature drug release and insufficient targeting efficiency, which hinder their clinical translation. The use of SPMs analogs with significantly extended half-lives could introduce risks of side effects. The sustained suppression of inflammatory responses by such long-acting compounds may impair the body’s ability to activate necessary defense mechanisms, whereas moderate inflammatory responses play a protective role in disease progression. Thus, a balance between efficacy and risk needs to be carefully considered, and further investigation is needed. Elucidating the mechanisms linking kidney dysfunction with abnormalities in SPMs biosynthesis could potentially allow therapeutic goals to be achieved by modulating endogenous SPMs levels. Due to the complexity of the pathological environment of kidney diseases and individual differences, the development of universal therapeutic strategies still requires extensive clinical and basic research validation. Nevertheless, this approach holds significant clinical value.

## 5 Conclusion

Unresolved inflammation serves as a critical determinant in the progression of renal failure and fibrosis. The process of neutrophil apoptosis represents a pivotal turning point between inflammatory resolution and progression, with SPMs emerging as promising novel targets for regulating neutrophil apoptosis and clearance, demonstrating substantial therapeutic potential in kidney diseases. While the dysregulation of inflammatory resolution mechanisms in kidney diseases presents new therapeutic opportunities for drug development, comprehensive research is essential to elucidate the underlying mechanisms, facilitate clinical investigations, and optimize therapeutic applications. Potential therapeutic strategies may include selective targeting, precise intracellular delivery of SPMs, and controlled induction of programmed neutrophil apoptosis to modulate immune homeostasis and inflammatory responses. The monitoring of SPM biomarkers and neutrophil functional status holds significant promise in evaluating disease severity, prognosis, and outcomes in kidney diseases. Moreover, these findings indicate that therapeutic approaches to inflammatory diseases should extend beyond conventional inhibitors to encompass agonists such as SPMs, which activate endogenous inflammatory resolution processes and establish a new homeostatic balance, potentially enabling earlier therapeutic intervention and mitigating chronic disease progression.
